# Facility and resident characteristics associated with variation in nursing home transfers: evidence from the OPTIMISTIC demonstration project

**DOI:** 10.1186/s12913-021-06419-y

**Published:** 2021-05-24

**Authors:** Justin Blackburn, Casey P. Balio, Jennifer L. Carnahan, Nicole R. Fowler, Susan E. Hickman, Greg A. Sachs, Wanzhu Tu, Kathleen T. Unroe

**Affiliations:** 1 Indiana University Richard M. Fairbanks School of Public Health, 1050 Wishard Blvd, RG 5194, Indianapolis, IN USA; 2 Cecil G. Sheps Center for Health Services Research, University of North Carolina, Chapel Hill, NC USA; 3 Indiana University School of Medicine, Indianapolis, IN USA; 4 Regenstrief Institute, Inc, Indianapolis, IN USA; 5 Indiana University School of Nursing, Indianapolis, IN USA; 6 Department of Biostatistics, Indiana University School of Medicine, Indianapolis, IN USA

**Keywords:** Nursing facility, Resident characteristics, Medicare, Long-term care, Avoidable hospitalizations

## Abstract

**Background:**

Centers for Medicare and Medicaid Services (CMS) funded demonstration project to evaluate financial incentives for nursing facilities providing care for 6 clinical conditions to reduce potentially avoidable hospitalizations (PAHs). The Optimizing Patient Transfers, Impacting Medical Quality, and Improving Symptoms: Transforming Institutional Care (OPTIMISTIC) site tested payment incentives alone and in combination with the successful nurse-led OPTIMISTIC clinical model. Our objective was to identify facility and resident characteristics associated with transfers, including financial incentives with or without the clinical model.

**Methods:**

This was a longitudinal analysis from April 2017 to June 2018 of transfers among nursing home residents in 40 nursing facilities, 17 had the full clinical + payment model (1726 residents) and 23 had payment only model (2142 residents). Using CMS claims data, the Minimum Data Set, and Nursing Home Compare, multilevel logit models estimated the likelihood of all-cause transfers and PAHs (based on CMS claims data and ICD-codes) associated with facility and resident characteristics.

**Results:**

The clinical + payment model was associated with 4.1 percentage points (pps) lower risk of all-cause transfers (95% confidence interval [CI] − 6.2 to − 2.1). Characteristics associated with lower PAH risk included residents aged 95+ years (− 2.4 pps; 95% CI − 3.8 to − 1.1), Medicare-Medicaid dual-eligibility (− 2.5 pps; 95% CI − 3.3 to − 1.7), advanced and moderate cognitive impairment (− 3.3 pps; 95% CI − 4.4 to − 2.1; − 1.2 pps; 95% CI − 2.2 to − 0.2). Changes in Health, End-stage disease and Symptoms and Signs (CHESS) score above most stable (CHESS score 4) increased the risk of PAH by 7.3 pps (95% CI 1.5 to 13.1).

**Conclusions:**

Multiple resident and facility characteristics are associated with transfers. Facilities with the clinical + payment model demonstrated lower risk of all-cause transfers compared to those with payment only, but not for PAHs.

**Supplementary Information:**

The online version contains supplementary material available at 10.1186/s12913-021-06419-y.

## Introduction

Long-term nursing home residents have an increased risk of transfers to the hospital including emergency department (ED) visits, observation stays, and hospital admissions [1–3]. Adverse consequences of hospitalizations have been well-documented among older adults, and include cognitive and/or functional decline, risk of iatrogenic disease, and added cost—over $14 billion dollars in 2011 [1, 4–6]. Furthermore, up to 60% of hospitalizations are considered potentially avoidable hospitalizations (PAHs), meaning transfer could have been prevented through early detection or improved management of the condition within the nursing home [7–9]. Although different methodologies are used to classify the avoidability of transfer, hospital discharge-based algorithms have determined congestive heart failure, pneumonia, urinary tract infections, sepsis, skin infections, and dehydration have a high probability of being PAHs [7, 8]. Several factors are associated with PAHs, including resident comorbidities (e.g. renal disease, diabetes), resident race (i.e. Black), lower facility staffing levels, and lower reimbursement rates [8, 10, 11]. A greater understanding of resident and facility characteristics is needed to guide efforts aimed at reducing resident transfers.

The Centers for Medicare and Medicaid Services (CMS) Innovation Center launched a clinical demonstration project in 2012 to test models to reduce PAHs [12]. Seven project sites implemented unique clinical models and the Indiana site was called Optimizing Patient Transfers, Impacting Medical Quality, and Improving Symptoms: Transforming Institutional Care (OPTIMISTIC). The OPTIMISTIC clinical model deployed registered nurses (RNs) and nurse practitioners (NPs) to participating CMS-certified nursing homes to provide targeted monitoring and assessment of long-term residents, staff training, and coordination with the primary care provider team. The OPTIMISTIC clinical model includes systematic advance care planning, root-cause analysis of all transfer events, support for rapid recognition of an acute change in status and then close follow-up for acutely ill residents managed in place. A robust, multi-site evaluation of the overall CMS initiative found that all-cause transfers and PAHs were reduced without increasing mortality [13–15]. Among participating facilities in Indiana from 2014 to 2016, 26.2% of OPTIMISTIC residents had an all-cause hospitalization and 11.8% had a PAHs, which were 5.1 and 3.9 percentage points lower than comparison residents, respectively [16].

A second phase began in October 2016 among six of the original CMS-funded sites, including OPTIMISTIC. As with the 19 facilities originally recruited and completed Phase 1, specific criteria were required of the 23 facilities recruited to join Phase 2, and described in greater detail elsewhere [17]. Notably, as a demonstration project, recruitment of facilities was not random. For example, in Phase 1, facilities were concentrated in the Indianapolis area to facilitate delivery of the in-person clinical model; recruitment of Phase 2 facilities was state-wide. However, all facilities demonstrated tools, processes, and services were in place to provide onsite care Phase 2 introduced special Medicare billing codes (Healthcare Common Procedure Coding System [HCPCS] codes G9679–G9684) enabling additional reimbursement for acute care provided onsite to participating facilities and medical providers (approximately $218 per day) [17]. Facilities could bill for six procedure codes corresponding to pneumonia, COPD/asthma, dehydration, congestive heart failure, skin infections, and urinary tract infections. Codes were used if residents experienced a change in condition, met condition-specific clinical criteria, and were certified by a medical provider for in-facility care up to 7 days. Facilities that participated in Phase 1 of OPTIMISTIC received billing codes in addition to the established clinical model, which continued in Phase 2; facilities joining in Phase 2 had access to the billing codes but did not implement the clinical model [17, 18].

Sizable differences were observed in hospitalization reductions among OPTIMISTIC facilities in Phase 1, indicating facility-level variation may explain some aspects of implementation or success [19]. However, additional analyses are needed to elucidate the source of this variation, including facility and resident characteristics. Furthermore, characteristics associated with the risk of transfers within the context of the financial incentives introduced in Phase 2 of the OPTIMISTIC Project may offer insights into successful implementation of new care and payment models.

## Methods

### Sample and setting

Although Phase 2 officially began in October 2016, this study focuses on eligible residents in participating nursing facilities between April 2017 and June 2018 allowing a 6-month learning period for facilities to implement processes for identifying resident changes in conditions which were eligible for the use of new billing codes. Eligible residents were defined by CMS as long-stay residents (> 100 days in facility) enrolled in Medicare Parts A and B fee-for-service (i.e. without Medicare Advantage). Residents were censored if they died or became ineligible because of discharge from the facility, enrolled in Medicare Advantage plan, or were admitted to hospice. Residents were analyzed based on eligibility periods designated by three-month quarters according to calendar year. Resident observations were included only for quarters in which they were eligible for at least 1 day during the quarter. Among 40 total facilities, 23 facilities were in the payment only group and 17 in the clinical + payment group.

### Data

Demographic and chronic condition information were derived from the Master Beneficiary Summary File (MBSF). Medicare claims data were used to identify all transfers, including the carrier line file, inpatient, and outpatient files. Data were supplemented by the Minimum Data Set (MDS) assessments for measures of health, functional, and cognitive status. Nursing homes are required to complete an MDS assessment on all residents at the time of admission and quarterly thereafter, or after a significant change in status. Thus, the most recent full MDS assessment was used to represent each resident-quarter in this study period. Quarter in which death occurred was identified from the MBSF or MDS. Nursing Home Compare data, including Provider Files for facility staffing and quality measures, and the Provider of Service (POS) Files for facility characteristics including rural status, for-profit status, and whether the facility was part of a chain.

### Outcomes

The two primary outcomes of focus for our analyses were all-cause transfers, including hospitalization, ED, or observation, and PAHs. Both are primary targets for reduction by the OPTIMISTIC clinical model and the financial incentive payments. However, to put our outcomes into context, we considered a variety of transfers consistent with the national, multi-site evaluation of the demonstration project, including all-cause ED visits, all-cause hospitalizations, PAHs, potentially avoidable EDs, hospitalization for any of the six conditions corresponding to financial incentive billing codes [18]. Those treated and discharged from the ED were considered an EDvisit, while admissions to the hospital, regardless of the route, were considered hospitalizations. Outcomes were not mutually-exclusive, such that PAHs were nested within all-cause hospitalizations. Outpatient and carrier files were used to identify ED visits with revenue center codes 450–459, or 981 that did not result in admission. Hospitalizations were any consecutive stays as in inpatient, regardless of whether transferred to another hospital during the stay. Observation stays were identified as claims with a revenue center code 0762 that lacked a corresponding overnight stay [20]. The identification of potentially avoidable for PAHs and ED visits was done using International Classification of Disease version 9 or 10 (ICD-9 / ICD-10) codes consistent with the larger evaluation of the demonstration project [18]. Due to the sparse data representing repeated transfers among residents within a quarter, counts of transfers were aggregated to binary indictors for whether at least one of each type of transfer occurred within a given quarter.

### Measures- resident & facility characteristics

Potential resident and facility characteristics were included based on a priori hypotheses informed from prior evidence and the strength of the bivariate associations [1, 3, 7, 11, 21–27]. Resident demographic and health status information included chronic conditions identified from the annually-updated MBSF Chronic Conditions Warehouse (CCW) segment including Alzheimer’s Disease and Related Dementia, chronic kidney disease, COPD, congestive heart failure, diabetes, ischemic heart disease, depression, osteoporosis, stroke/transient ischemic attack, and hypertension. Other health status measures were derived quarterly from the MDS including cognitive functioning scale (CFS) (range cognitively intact [1]-severe impairment [4]), Changes in Health, End-stage disease and Symptoms and Signs (CHESS) score (range most stable [0]-least [5]), and activities of daily living (ADL) (range complete independence [0]-complete dependence on staff [28]) [21–23]. A small number of residents (*n* = 5) were excluded because ADL, CFS, and/or CHESS score could not be calculated based on the completeness of the data available their MDS assessment. Addtionally, because only a small number of eligible residents were recorded with the least stable CHESS score, they were excluded (*n* = 12).

Facility characteristics included an indicator for which OPTIMISTIC intervention the facility received: payment only model versus the full clinical + payment model. Additionally, from the Nursing Home Compare Provider Files, quarterly categorical indicators for staffing (total licensed hours per resident per day), bed size, and overall star rating (1–5) for the last month of the quarter. Binary indicators for rural setting, for-profit/non-profit status, and chain-owned were obtained from the CMS POS files at baseline (December 2017).

### Analysis

We used mixed-effects logistic regression to account for repeated resident observations over time and nesting within facilities. This multi-level approach has been used previously when resident nesting within facilities occurs [27–29].Our modeling strategy estimated residents’ outcome risk over time for each characteristic accounting for all other resident and facility characteristics. Furthermore, we estimate the amount of variance in the outcome attributed to the resident and facility, and estimate the association of resident and facility characteristics using a similar modeling strategy as Herrin et al. [30] The intraclass correlation coefficient for null multi-level model was compared to the full model (with covariates) to quantify the variance explained at each level and by the addition of resident and facility characteristics. Marginal effects were estimated and reported with 95% confidence intervals, which can be interpreted as the absolute risk different in the outcome holding all other characteristics at the mean, expressed as a percentage point change. Interactions were considered for facilities in the payment only group or the clinical + payment group to determine whether the OPTIMISTIC model or financial incentives only modified the risk of transfer. Variables which demonstrated weak bivariate associations were not included in the final models.

## Results

During the study period, 3868 unique residents in OPTIMISTIC facilities contributed 12,787 resident-quarter observations. At least one transfer was observed during 2290 (17.9%) resident-quarters. Among them, we observed 1439 hospitalizations (11.2% of resident-quarters), 1204 all-cause ED visits (9.4% of resident-quarters), 487 PAHs 487 (3.8% of resident-quarters), 308 hospitalizations for any of the six conditions (2.4% of resident-quarters) and 256 potentially avoidable ED visits (2.0% of resident-quarters).

**Fig. 1:**
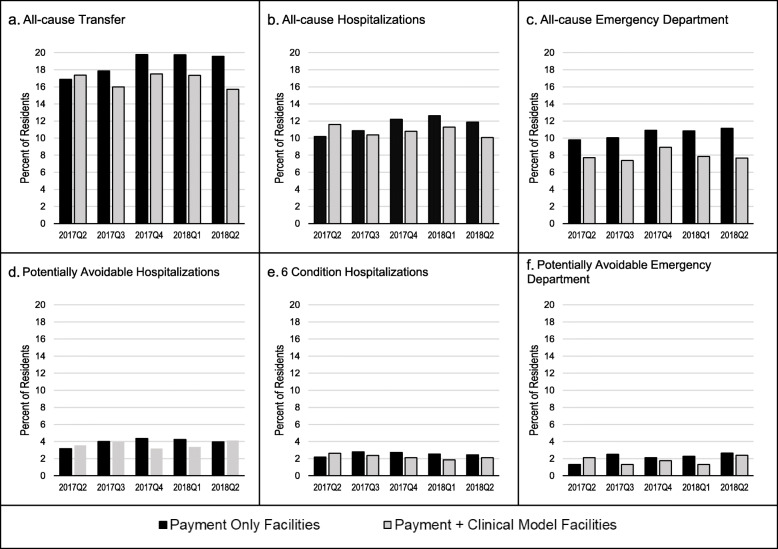
Observed percent of OPTIMISTIC nursing home residents experiencing at least one of each transfer type by quarter, stratified by payment only facilities and clinical + payment. Note: All-cause transfers include hospitalizations, emergency department (ED) visits, and observation stays. ED visits that ended in a hospitalization are included in all-cause transfers and hospitalizations but not in ED visits. The 6 conditions include pneumonia, COPD/asthma, dehydration, congestive heart failure, skin infections, and urinary tract infections

Figure 1 displays the percentage of residents with at least one transfer by each type over time, stratified by intervention group. All-cause transfers increased over time among residents in payment only facilities, ranging from 16.8% in the first quarter to 19.6% 1 year later, while clinical + payment facilities remained stable, and consistently lower, at approximately 17% of residents. Similar differences were observed for all-cause ED visits and all-cause hospitalizations. The percent of residents with PAHs ranged between 3 and 4% for the duration of the study period, with no apparent difference by intervention group.

Residents were mostly female (69.2%), white (84.8%), Medicare-Medicaid dual-eligible (75.1%), with some form of Alzheimer’s Disease/Dementia (81.3%) and depression (74.5%, Table 1). On average, residents contributed 2.5 quarters during the study period (standard deviation [SD] ±1.5) and 907 (7.1%) residents died (average eligibility 2.6 quarters, SD ±1.3). Fifty-seven percent of the resident-quarter observations were in payment only facilities and 43% in payment + clinical facilities (Table 2). Bivariate associations were observed with staffing ratios, resident gender, Medicare-Medicaid dual eligibility status, CFS, CHESS, marital status, and multiple individual chronic conditions with both all-cause and PAHs (*p* < 0.05 for each). However, some notably race, ADL score, facility intervention group, and for-profit status were associated with all-cause transfer only (*p* < 0.05 for each).

**Table 1:** Frequencies of individual-level characteristics (*N* = 12,787 resident-quarters) as well as bivariate statistics for all-cause transfer (*n* = 2290) and potentially avoidable hospitalizations (*n* = 487)

	Overall (*N* = 12,787)	Payment + Clinical Model Facilities (*n* = 5555)	All-cause Transfer (*n* = 2290)		Potentially Avoidable Hospitalization (*n* = 487)	
	%	%	%	*P*	%	*P*
Resident-Quarters by Time Period						
April–June 2017	21.0	20.8	20.0	0.269	18.3	0.672
July–September 2017	20.4	20.3	19.4		21.4	
October–December 2017	20.3	20.2	21.3		20.5	
January–March 2018	19.4	19.5	20.3		19.7	
April–June 2018	19.0	19.2	19.0		20.1	
Age						
≤ 64 years	9.9	9.1	15.9	< 0.001	15.4	< 0.001
65–74 years	16.6	14.8	19.1		20.3	
75–84 years	28.7	28.0	29.7		33.3	
85–94 years	35.8	38.2	29.5		26.9	
≥ 95 years	9.0	10.0	5.7		4.1	
Gender						
Male	30.8	29.1	37.0	< 0.001	37.4	0.001
Female	69.2	70.9	63.0		62.6	
Race/ethnicity						
White	84.8	93.7	82.6	0.001	81.1	0.067
Black	13.7	5.2	16.1		17.3	
Other	1.5	1.1	1.3		1.6	
Medicare-Medicaid Dual Eligibility						
Medicare Eligible Only	24.9	28.0	29.6	< 0.001	33.5	< 0.001
Dual Eligible	75.1	72.0	70.4		66.5	
Cognitive Functioning Scale (CFS)						
CFS 1	30.9	32.2	39.0	< 0.001	40.7	< 0.001
CFS 2	22.9	24.3	24.5		25.5	
CFS 3	38.9	37.7	30.7		30.8	
CFS 4	7.3	5.8	5.9		3.1	
Changes in Health, End-stage disease and Symptoms and Signs (CHESS)						
0	35.2	41.6	28.8	< 0.001	23.6	< 0.001
1	34.7	33.7	34.3		34.5	
2	22.7	19.2	26.1		29.9	
3	6.4	4.7	9.1		10.7	
4	1.0	0.8	1.8		2.3	
Activities of Daily Living (ADLs)						
1st Quartile (≤ 17)	29.6	36.8	27.6	0.010	28.1	0.479
2nd Quartile (18–19)	25.1	26.4	26.4		27.5	
3rd Quartile (20–21)	32.7	25.9	31.9		30.8	
4th Quartile (22–28)	12.6	10.9	14.1		13.6	
Presence of Chronic Conditions						
Acute Myocardial Infarction	9.2	8.4	12.6	< 0.001	16.2	< 0.001
Alzheimer’s Disease / Dementia	81.3	80.1	75.2	< 0.001	77.6	0.035
Chronic Kidney Disease	57.9	56.4	68.0	< 0.001	71.5	< 0.001
Chronic Obstructive Pulmonary Disease	42.1	42.5	52.0	< 0.001	57.3	< 0.001
Congestive Heart Failure	56.7	58.0	63.3	< 0.001	68.6	< 0.001
Diabetes	50.1	49.9	56.2	< 0.001	61.4	< 0.001
Ischemic Heart Disease	62.9	63.1	66.6	< 0.001	73.1	< 0.001
Depression	74.5	73.0	76.1	0.050	78.4	0.042
Osteoporosis	38.0	38.8	32.7	< 0.001	30.6	0.001
Stroke / Transient Ischemic Attack	36.3	36.0	38.6	0.012	41.1	0.026
Hypertension	89.3	89.0	88.2	0.046	87.5	0.176
Never Married	13.1	12.7	14.6	< 0.001	14.8	0.014
Married	20.8	21.0	21.5		23.8	
Widowed	44.5	48.8	40.5		37.4	
Not Married	21.6	17.5	23.4		24.0	

Results are provided for the resident-quarter level. Residents were included in the analyses if they were eligible for at least 1 day of the quarter. On average, residents contributed 2.5 quarters during the study period (standard deviation [SD] ±1.5) and 907 (7.1%) residents died (average eligibility 2.6 quarters, SD ±1.3). Results provide descriptions of the population by the percent of resident-quarter observations for given characteristics, and by residents experiencing at least one all-cause transfer or PAH during a given quarter

*Abbreviations*: *CHESS* Changes in Health, End-stage disease and Symptoms and Signs, *ADLs* Activities of Daily Living

**Table 2:** Frequencies of facility-level characteristics (*N* = 12,787 resident-quarters) as well as bivariate statistics for all-cause transfer (*n* = 2290) and potentially avoidable hospitalizations (*n* = 487)

	Overall (*N* = 12,787)	Payment + Clinical Model Facilities (*n* = 5555)	All-cause Transfer (*n* = 2290)		Potentially Avoidable Hospitalization (*n* = 487)	
	%	%	%	*P*	%	*P*
Payment Only Facility	56.6	–	59.3	0.004	58.7	0.325
Payment + Clinical Model Facility	43.4	100	40.7		41.3	
Staffing Ratio (NHPPD)						
1st Quartile (≤ 3.31)	23.8	16.4	25.8	< 0.001	26.7	0.004
2nd Quartile (3.32–3.55)	24.3	25.7	24.1		23.4	
3rd Quartile (3.56–4.15)	24.9	25.8	27.2		29.2	
4th Quartile (≥ 4.15)	27.1	32.2	22.9		20.7	
Number of Facility Beds						
1st Quartile (≤ 115)	23.9	30.1	26.4	< 0.001	26.1	0.003
2nd Quartile (116–147)	27.5	31.9	28.8		27.5	
3rd Quartile (148–169)	23.8	11.2	23.9		28.1	
4th Quartile (≥ 170)	24.8	26.8	20.9		18.3	
Overall CMS Star Rating						
1 Star	4.5	7.1	8.2	< 0.001	7.0	0.022
2 Stars	10.4	7.5	12.2		12.1	
3 Stars	20.7	11.5	20.0		17.3	
4 Stars	28.5	29.1	26.9		28.1	
5 Stars	35.9	44.8	32.7		35.5	
Rural-Urban Location						
Urban	72.8	51.8	73.5	0.367		
Rural	27.2	48.1	26.5		30.4	0.109
For-profit Status						
For-profit Facility	15.7	16.8	17.6	0.004	16.0	0.820
Not for-profit Facility	84.4	83.3	82.4			
Multi-facility Ownership	76.3	71.0	78.7	0.002	80.5	0.026

Results are provided for the resident-quarter level. Residents were included in the analyses if they were eligible for at least 1 day of the quarter. Results provide descriptions of the population by the percent of resident-quarter observations for given characteristics and by residents experiencing at least one all-cause transfer or PAH during a given quarter

*Abbreviations*: *NHPPD* nursing hours per patient day

The absolute risk differences of characteristics associated with all-cause transfers and PAHs, estimated using multilevel logit regression, are shown in Table 3. Notable characteristics associated with PAH risk include residents aged 95 years or older (− 2.4 percentage points [pps]; 95% CI − 3.8 to − 1.1), history of hypertension (− 3.7 pps; 95% CI − 5.2 to − 2.1), Medicare-Medicaid dual-eligibility (− 2.5 pps; 95% CI − 3.3 to − 1.7), advanced cognitive impairment (CFS category 4) (− 3.3 pps; 95% CI − 4.4 to − 2.1) and moderate impairment (CFS category 3) (− 1.2 pps; 95% CI − 2.2 to − 0.2). Higher CHESS scores relative to the most stable were associated with increased risk of PAH including CHESS score of 4 (7.3 pps; 95% CI 1.5 to 13.1), CHESS score 3 (4.3 pps; 95% CI 2.0 to 6.6), and CHESS score 2 (2.4 pps; 95% CI 1.2 to 3.5). Each characteristic was also associated with all-cause transfer risk.

**Table 3:** Estimated risk difference for characteristics associated with resident transfers (*N*=12,787 resident-quarters)

	All-cause Transfer (***n***=2,290)		Potentially Avoidable Hospitalization (***n***=487)	
	Marginal Effects	95% CI	Marginal Effects	95% CI
Individual-level Characteristics				
Aged ≤ 64 years	8.6	(5.0, 12.2)	1.5	(-0.2, 3.3)
Aged 65-74 years	Reference			
Aged 75-84 years	-1.0	(-3.5, 1.5)	0.1	(-1.1, 1.3)
Aged 85-94 years	-4.5	(-7.1, -2.0)	-1.4	(-2.5, -0.2)
Aged ≥ 95 years	-7.7	(-10.9, -4.4)	-2.4	(-3.8, -1.1)
Female	-2.6	(-4.4, -0.8)	-0.1	(-0.9, -0.7)
White	Reference			
Black	3.4	(0.9, 6.0)	1.3	(0.1, 2.6)
Other	-4.6	(-9.9, 0.8)	0.3	(-2.6, 3.3)
Dual Eligible	-6.7	(-8.4, -5.1)	-2.5	(-3.3, -1.7)
Cognitive Functioning Scale 1	Reference			
Cognitive Functioning Scale 2	-0.8	(-2.9, 1.3)	-0.3	(-1.4, 0.8)
Cognitive Functioning Scale 3	-4.5	(-6.6, -2.5)	-1.2	(-2.2, -0.2)
Cognitive Functioning Scale 4	-6.9	(-10.1, -3.8)	-3.3	(-4.4, -2.1)
CHESS Score 0	Reference			
CHESS Score 1	1.9	(0.1, 3.6)	1.1	(0.3, 1.9)
CHESS Score 2	4.7	(2.4, 7.0)	2.4	(1.2, 3.5)
CHESS Score 3	9.3	(5.4, 13.1)	4.3	(2.0, 6.6)
CHESS Score 4	13.8	(5.6, 22.0)	7.3	(1.5, 13.1)
ADLs 1st Quartile (≤ 17)	Reference			
ADLs 2nd Quartile (18-19)	3.8	(1.9, 5.8)	0.8	(-0.2, 1.8)
ADLs 3rd Quartile (20-21)	2.6	(0.6, 4.6)	-0.4	(-1.3, 0.6)
ADLs 4th Quartile (22-28)	4.1	(1.1, 7.0)	-0.1	(-1.4, 1.3)
Acute Myocardial Infarction	3.2	(0.6, 5.8)	1.3	(0.2, 2.4)
Alzheimer’s Disease / Dementia	-3.0	(-5.2, -0.7)	0.4	(-0.1, 1.5)
Chronic Kidney Disease	6.2	(4.3, 8.0)	1.6	(0.6, 2.5)
Chronic Obstructive Pulmonary Disease	4.6	(2.8, 6.3)	1.0	(0.2, 1.9)
Ischemic Heart Disease	2.2	(0.2, 4.1)	1.3	(0.3, 2.3)
Hypertension	-6.9	(-10.1, -3.7)	-3.7	(-5.2, -2.1)
Facility-level Characteristics				
Payment Only Facility	Reference			
Payment + Clinical Model Facility	-4.1	(-6.2, -2.1)	-0.5	(-1.6, 0.4)
Beds 1st Quartile (≤ 115 beds)	Reference			
Beds 2nd Quartile (116 – 147 beds)	-3.4	(-5.6, -1.1)	-0.9	(-1.9, 0.2)
Beds 3rd Quartile (148 – 169 beds)	-0.2	(-2.7, 2.2)	0.7	(-0.5, 0.2)
Beds 4th Quartile (≥ 170 beds)	-3.4	(-6.0, -0.1)	-0.9	(-2.1, 0.3)
Overall Star Rating 1	13.0	(8.3, 17.6)	2.4	(-0.2, 4.9)
Overall Star Rating 2	4.6	(1.7, 7.4)	0.6	(-0.9, 2.1)
Overall Star Rating 3	1.7	(-0.5, 3.9)	-0.7	(-1.7, 0.3)
Overall Star Rating 4	0.4	(-1.5, 2.3)	-0.2	(-1.2, 0.7)
Overall Star Rating 5	Reference			
2017 Quarter 2	Reference			
2017 Quarter 3	1.3	(-0.7, 3.2)	1.2	(0.2, 2.1)
2017 Quarter 4	2.5	(0.5, 4.5)	0.9	(-0.1, 1.9)
2018 Quarter 1	3.2	(1.1, 5.2)	1.1	(0.1, 2.1)
2018 Quarter 2	1.9	(-0.3, 4.0)	1.2	(0.1, 2.3)

Model also controls for residents with congestive heart failure, diabetes, ischemic heart disease, depression, osteoporosis, stroke / transient ischemic attack and marital status, as well as facility characteristics staffing level, rural status, for-profit status, and multi-facility ownership

*Abbreviations*: *CI* confidence interval, *ADLs* Activities of Daily Living, *NHPPD* nursing hours per patient day

Notable resident characteristics associated with all-cause transfers but not PAHs included ADL scores above 17 (most independent) including ADL 18–19 (3.8 pps; 95% CI 1.9 to 5.8), ADL 20–21 (2.6 pps; 95% CI 0.6 to 4.6), ADL 22–28 (4.1 pps; 95% CI 1.1 to 7.0) and residents with Alzheimer’s Disease/dementia (− 3.0 pps; 95% CI − 5.2 to − 0.7). Notable facility characteristics associated with all-cause transfers, but not PAHs, included the presence of the clinical + payment model (− 4.1 pps; 95% CI − 6.2 to − 2.1), 116 to 147 beds in the facility (− 3.4 pps; 95% CI − 5.6 to − 1.1), and overall star rating of 1 (13.0 pps; 95% CI 8.3 to 17.6) and overall star rating of 2 (4.6 pps; 95% CI 1.7 to 7.4).

From the intraclass correlation coefficient estimates, we observed 20% of the variance in all-cause transfers was explained by residents within the same facilities (i.e. at the facility level) and 32% by residents over time (i.e. at the resident-level). Similar levels of nesting were observed for PAHs, with 20% of variance explained at the facility-level and 35% at the resident-level. Introducing covariates reduced the unexplained variance at the facility-level by approximately 10 pps and at the resident-level by approximately 6 pps (7 pps and 13 pps reductions among PAHs, respectively). Resident and facility characteristics demonstrated heterogeneity among resident-quarters by clinical + payment or payment only facilities including race, CHESS Scale, and bed size. Although statistical evidence of interaction for the presence of the clinical + payment model was not definitive, stratified results are presented in the Additional file 1.

## Discussion

In facilities participating in a demonstration project to reduce PAHs, we identified age, Medicare-Medicaid dual-eligibility, cognitive function, CHESS, and history of certain comorbidities were associated with the risk of all-cause transfer, including PAHs. These characteristics generally reflect limitations to residents’ functional status and a history of debilitating comorbidities. However, some characteristics were inconsistently associated with the outcomes. Resident ADL score, history of Alzheimer’s Disease/dementia, CMS Star Rating, facility bed count, and participation in the clinical + payment model was associated with the risk of all-cause transfer, but not with the risk of PAHs. Although variation in the risk of transfer was explained at both the resident- and facility-level, resident characteristics explained a greater percentage. These results add to the existing knowledge regarding how to reduce transfers, specifically PAHs, among nursing home residents. Furthermore, risk stratification of nursing home residents by specific characteristics may be necessary for consideration in planning and evaluation of future efforts to reduce PAHs.

The oldest residents (≥ 95 years), the most cognitively impaired (CFS 4), and Medicare-Medicaid dual-eligible residents had the lowest adjusted risk of both all-cause transfers and PAHs. The lower risk of hospitalization for older residents is equivocal in other studies and may be dependent on how well functional status and comorbidities are measured and controlled for [24]. Lower risk in the oldest age group may be attributable to informative censoring, wherein residents in this age group remaining eligible for OPTIMISTIC are overall healthier than those who lose eligibility due to hospice enrollment. To avoid this, some studies exclude observations within 12 months of residents’ death [11]. However, due to concerns about sample size reductions and the importance of including events that will enhance our understanding of PAHs, this approach was not feasible. Alternatively, resident characteristics may be confounded by completion of advance care planning/advance directives wherein transfer is inconsistent with their goals of care. Residents in clinical + payment facilities had consistent access to structured Advance Care Planning through the OPTIMISTIC nurses as this is a key part of the clinical care model. OPTIMISTIC nurses were trained in Advance Care Planning conversations and this is a dedicated component of the role [31]. All facilities had access to tailored educational materials related to palliative care and incorporating goals of care into treatment plans. This may also explain the lower risk of transfer among cognitively impaired residents, and is consistent with other studies of residents with advanced illness and limited life expectancy [25]. Although residents with advanced age, cognitive impairment, and/or comorbidities may have clinical features consistent with hospital-level care, decisions to transfer are complex and mitigated by multiple factors that are not captured by the MDS [25, 32–34].

In contrast to lower risk of transfer for the oldest residents, residents in the youngest age category—those under 64 years old and thus qualifying for Medicare based on disability—had an increased risk of all-cause transfers, but not PAHs. This finding is consistent with another study where younger age also had functional impairment [35, 36]. Furthermore, the lack of an association with PAHs may indicate that hospitalizations among younger residents tend not to be considered potentially avoidable. Moreover, risk of PAHs may not be uniform across all age groups and warrants further investigation.

We observed an increased risk of transfer with higher CHESS Scale scores, which may seem counter-intuitive. One potential explanation is that the CHESS Scale reflects recent health instability, some of which may appear treatable [37]. Although CHESS Scale is a predictor of mortality, it may also be sensitive to acute resident changes resulting in transfers, including acute mental status change, dehydration, and pressure ulcers [21]. Furthermore, there may be multicollinearity with CHESS Scale and other resident characteristics included in our model, including cognitive impairment. However, variance inflation factors from our models were less than 3 for all variables included.

Facility characteristics including bed size, CMS Overall Star Rating, and presence of the clinical + payment model were associated with reduced all-cause transfers, however the associations were not statistically significant with PAHs. Nationally, the overall risk of hospital transfers among nursing home residents has decreased since 2011, coinciding with multiple initiatives to reduce hospitalizations [25]. Other policies, such as the introduction of value-based pursing to the skilled nursing prospective payment system beginning in 2018 were intended to improve quality of care within facilities, although it is not known whether any improvements affect the transfer risk among long-stay residents [38]. Previous analyses from the OPTIMISTIC project**,** which has similar components as the INTERACT Program, demonstrated benefits to improving the management of acute changes in residents’ condition and observed reductions in transfers [13, 14, 17, 19, 31, 39]. The optimal level or mix of incentives (or penalties) sufficient to drive investments in clinical care models and other needed resources to provide high quality care in place remains an important question.

Although we do not observe a decrease in transfer risk during our study period, residents in OPTIMISTIC clinical + payment model facilities maintained a consistently lower risk of all-cause transfer than payment only facilities, which increased slightly over time. The OPTIMISTIC clinical model was first introduced in 2012. Thus, nearly half of the facilities represented in our analyses were focused on reducing transfers, specifically PAHs, using specially trained nurses for 4 years prior to the release of the payment reimbursement codes. The payment component was introduced in the fourth quarter of 2016 and thus the payment only facilities may have had a more heterogeneous experience reducing PAHs [31]. Furthermore, the payment only facilities lacked on-site nurses trained to detect and treat conditions to reduce PAHs, instead relevant staff were trained on the use of billing codes for the six conditions.

The absence of an association between PAHs and the clinical + payment model is notable. This may be attributable to limitations of the identification of PAHs from claims data. First, we cannot exclude the possibility that coding practices designed to maximize hospital reimbursement may affect whether a hospitalization was subsequently determined to be a PAH; it is known that administrative claims data provides only a partial picture of the actual clinical experience [40]. Furthermore, claims data do not provide direct insights into the primary reason for a transfer—this may be different than what was determined as the final principal diagnosis code for the hospital stay. Avoidability of transfers is difficult to assess, even by nursing staff within the facility, adding to the challenge of making a post-discharge determination using claims-based algorithms [17, 41, 42]. Finally, claims diagnoses reflect the full clinical stay which may include infections or other events that were not present on admission. Nonetheless, our findings that facilities with the clinical + payment model had lower rates of transfers highlights the importance of increasing capacity of facilities to treat patients in place with a proven clinical model such as OPTIMISTIC, and to support practice changes in addition to payment reform.

Although facility characteristics explained a substantial portion of the variation in transfer risk, we observed few with statistical associations. Among them, lower CMS Star Ratings were associated with increased transfers. Notably, initial recruitment of new facilities in Phase 2 of the OPTIMISTIC demonstration project required a minimum 3-star rating. However, facility rating fluctuates quarterly and maintenance was not a requirement, therefore we observed variation within participating facilities over time. It is possible that a decreasing Star Rating over time is indicative of decreasing overall quality of care.

This study has some limitations. First, we recognize this is a non-randomized intervention in 40 facilities within a single state. The lack of randomization could introduce selection bias and limit the generalizability of our results. Specifically, the selection of facilities to the clinical + payment group was based on several important characteristics, including a minimum CMS Star Rating, and were located in largely urban and suburban Central Indiana, may have resulted in bias as compared to the payment only group. We lack a true control group and instead used individuals’ risk over time to account for within-person confounding as well as differences in the intervention facility to contrast risk. The lack of control for some potentially important variables, such as the presence of newly diagnosed terminal diseases, family/resident preferences, or other relevant unmeasurable variables, could result in omitted variable bias. In such a case, the attribution of unobserved factors such as a do not resuscitate orders could bias the associations of other characteristics. We note this as an alternative explanation for the lower risk of transfer among residents with advanced cognitive impairment. However, prior work within this same demonstration project found that associations between advance care planning and potentially avoidable hospitalizations were attenuated after accounting for facility clustering and resident characteristics [31].

## Conclusions

Overall, our study contributes to a growing body of evidence around facility and resident characteristics associated with risk of transfers. In particular, we observe considerable variation at the facility-level, although few of the facility variables included were associated with resident transfers. However, facilities with the OPTIMISTIC clinical + payment model in place were able to demonstrate a lower risk of all-cause transfers as compared to those with payment only. We also raise questions about the ability to assess PAHs from claims data. Nonetheless, our findings provide evidence for risk stratification for facilities in reducing transfers and also highlight the potential benefit of additional staff in a proven clinical model supporting efforts to limit transfers for some potentially avoidable transfers.

## Supplementary Information

**Additional file 1: Appendix 1.** Overall cohort description (*N* = 12,787 resident-quarters) stratified by clinical + payment and payment only facilities. **Appendix 2.** Estimated risk difference for characteristics associated with all-cause resident transfers, stratified by clinical + payment and payment only facilities. **Appendix 3.** Estimated risk difference for characteristics associated with potentially avoidable hospitalizations, stratified by clinical + payment and payment only facilities.

## Data Availability

Limited versions of the datasets used and analyzed during the current study are available from the corresponding author on reasonable request and subject to approval from the sponsor.
