# Supplementary material for: Facility and resident characteristics associated with variation in nursing home transfers: evidence from the OPTIMISTIC demonstration project
Source: BMC Health Serv Res. 2021 May 24;21:492. doi: 10.1186/s12913-021-06419-y (PMC8142645; doi:10.1186/s12913-021-06419-y)
Supplement: Supplementary file 1 — Additional file 1: Appendix 1. Overall cohort description (N = 12,787 resident-quarters) stratified by clinical + payment and payment only facilities. Appendix 2. Estimated risk difference for characteristics associated with all-cause resident transfers, stratified by clinical + payment and payment only facilities. Appendix 3. Estimated risk difference for characteristics associated with potentially avoidable hospitalizations, stratified by clinical + payment and payment only facilities. [file 12913_2021_6419_MOESM1_ESM.docx]

**Supplementary Information**

**Facility and resident characteristics associated with variation in nursing home transfers: evidence from the OPTIMISTIC demonstration project**

Justin Blackburn PhD^a^, Casey P. Balio PhD^a^, Jennifer L. Carnahan MD, MPH^b,c^, Nicole R. Fowler, PhD, MHSA^b,c^, Susan E. Hickman PhD^b,c,d^, Greg A. Sachs, MD^b,c^, Wanzhu Tu PhD^e^, Kathleen T. Unroe MD, MHA^b,c^

Appendix 1. Overall cohort description (N=12,787 resident-quarters) stratified by clinical + payment and payment only facilities.

Appendix 2. Estimated risk difference for characteristics associated with all-cause resident transfers, stratified by clinical + payment and payment only facilities.

Appendix 3. Estimated risk difference for characteristics associated with potentially avoidable hospitalizations, stratified by clinical + payment and payment only facilities

Appendix 1. Overall cohort description (N=12,787 resident-quarters) stratified by clinical + payment and payment only facilities

| Individual-level Characteristics |  | Clinical + Payment  (5,555 resident-quarters) | Payment Only  (7,232 resident-quarters) |
| --- | --- | --- | --- |
|  |  | % | % |
|  | Aged ≤ 64 years | 10.9 | 9.1 |
|  | Aged 65-74 years | 18.9 | 14.8 |
|  | Aged 75-84 years | 29.7 | 28.0 |
|  | Aged 85-94 years | 32.7 | 38.2 |
|  | Aged ≥ 95 years | 7.8 | 10.0 |
|  | Male | 33.1 | 29.1 |
|  | Female | 66.9 | 70.9 |
|  | White | 73.1 | 93.7 |
|  | Black | 24.8 | 5.2 |
|  | Other | 2.0 | 1.1 |
|  | Medicare Eligible Only | 20.9 | 28.0 |
|  | Dual Eligible | 79.1 | 72.0 |
|  | Cognitive Functioning Scale 1 | 29.3 | 32.2 |
|  | Cognitive Functioning Scale 2 | 21.0 | 24.3 |
|  | Cognitive Functioning Scale 3 | 40.4 | 37.7 |
|  | Cognitive Functioning Scale 4 | 9.3 | 5.8 |
|  | CHESS Score 0 | 26.9 | 41.6 |
|  | CHESS Score 1 | 36.1 | 33.7 |
|  | CHESS Score 2 | 27.2 | 19.2 |
|  | CHESS Score 3 | 8.5 | 4.7 |
|  | CHESS Score 4 | 1.3 | 0.8 |
|  | ADLs 1st Quartile (≤ 17) | 20.3 | 36.8 |
|  | ADLs 2nd Quartile (18-19) | 23.4 | 26.4 |
|  | ADLs 3rd Quartile (20-21) | 41.5 | 25.9 |
|  | ADLs 4th Quartile (22-28) | 14.8 | 10.9 |
|  | Acute Myocardial Infarction | 10.3 | 8.4 |
|  | Alzheimer’s Disease / Dementia | 82.8 | 80.1 |
|  | Chronic Kidney Disease | 60.0 | 56.4 |
|  | Chronic Obstructive Pulmonary Disease | 41.7 | 42.5 |
|  | Congestive Heart Failure | 55.1 | 58.0 |
|  | Diabetes | 50.5 | 49.9 |
|  | Ischemic Heart Disease | 62.6 | 63.1 |
|  | Depression | 76.4 | 73.0 |
|  | Osteoporosis | 37.0 | 38.8 |
|  | Stroke / Transient Ischemic Attack | 36.8 | 36.0 |
|  | Hypertension | 89.7 | 89.0 |
|  | Never Married | 13.5 | 12.7 |
|  | Married | 20.5 | 21.0 |
|  | Widowed | 38.9 | 48.8 |
|  | Not Married | 27.0 | 17.5 |
| Facility-level Characteristics | Staffing 1st Quartile (≤ 3.31 NHPPD) | 33.5 | 16.4 |
|  | Staffing 2nd Quartile (3.32 – 3.55 NHPPD) | 22.4 | 25.7 |
|  | Staffing 3rd Quartile (3.56 – 4.15 NHPPD) | 23.7 | 25.8 |
|  | Staffing 4th Quartile (≥ 4.15 NHPPD) | 20.4 | 32.2 |
|  | Beds 1st Quartile (≤ 115 beds) | 15.8 | 30.1 |
|  | Beds 2nd Quartile (116 – 147 beds) | 21.9 | 31.9 |
|  | Beds 3rd Quartile (148 – 169 beds) | 40.2 | 11.2 |
|  | Beds 4th Quartile (≥ 170 beds) | 22.1 | 26.8 |
|  | Overall Star Rating 1 | 1.1 | 7.1 |
|  | Overall Star Rating 2 | 14.1 | 7.5 |
|  | Overall Star Rating 3 | 32.7 | 11.5 |
|  | Overall Star Rating 4 | 27.7 | 29.1 |
|  | Overall Star Rating 5 | 24.4 | 44.8 |
|  | Rural | 0 | 48.1 |
|  | For-profit Facility | 14.22 | 16.8 |
|  | Multi-facility Ownership | 83.1 | 71.0 |
| Transfers | All-cause Transfer | 16.8 | 18.7 |
|  | All-cause Hospitalization | 10.8 | 11.6 |
|  | All-cause Emergency Department Visit | 7.9 | 10.5 |
|  | Potentially Avoidable Hospitalization | 3.6 | 3.9 |
|  | Any 6 Condition Hospitalization | 2.2 | 2.5 |
|  | Potentially Avoidable Emergency Department Visit | 1.8 | 2.2 |
| Abbreviations: CHESS = Changes in Health, End-stage disease and Symptoms and Signs; ADLs = Activities of Daily Living; NHPPD = nursing hours per patient day  Note: Results are provided for the resident-quarter level. Residents were included in the analyses if they were eligible for at least one day of the quarter. Results provide descriptions of the population by the percent of resident-quarter observations for given characteristics, and by residents experiencing at least one all-cause transfer or PAH during a given quarter. | | | |

Appendix 2. Estimated risk difference for characteristics associated with all-cause resident transfers, stratified by clinical + payment and payment only facilities.

|  |  | **All-cause Transfer (2,290)** | | | |
| --- | --- | --- | --- | --- | --- |
|  |  | **Clinical + Payment (n=5,555 resident-quarters)** | | **Payment Only**  **(n=7,232 resident-quarters)** | |
| Individual-level Characteristics | Aged ≤ 64 years | 7.2 | (3.4, 11.1) | 7.8 | (2.9, 12.8) |
|  | Aged 65-74 years | Reference |  |  |  |
|  | Aged 75-84 years | -1.4 | (-4.7, 1.8) | -0.6 | (-4.0, 2.9) |
|  | Aged 85-94 years | -7.1 | (-10.7, -3.5) | -2.5 | (-6.1, 1.1) |
|  | Aged ≥ 95 years | -7.7 | (-13.2, -2.2) | -7.6 | (-11.9, -3.2) |
|  | Female | -2.0 | (-4.6, 0.6) | -2.9 | (-5.4, -0.5) |
|  | White | Reference |  |  |  |
|  | Black | 2.4 | (-0.3, 5.2) | 7.4 | (2.0, 12.7) |
|  | Other | -4.6 | (-13.0, 3.9) | -5.7 | (-13.6, 2.3) |
|  | Dual Eligible | -6.8 | (-9.3, -4.3) | -6.5 | (-8.7, -4.3) |
|  | Cognitive Functioning Scale 1 | Reference |  |  |  |
|  | Cognitive Functioning Scale 2 | -2.0 | (-5.1, 1.0) | -0.3 | (-3.2, 2.5) |
|  | Cognitive Functioning Scale 3 | -1.2 | (-4.2, 1.8) | -7.6 | (-10.3, -4.8) |
|  | Cognitive Functioning Scale 4 | -4.2 | (-9.2, 0.7) | -10.0 | (-14.3, -5.8) |
|  | CHESS Score 0 | Reference |  |  |  |
|  | CHESS Score 1 | 2.6 | (-0.4, 5.5) | 1.4 | (-0.9, 3.7) |
|  | CHESS Score 2 | 3.2 | (-0.3, 6.6) | 6.9 | (3.6, 10.1) |
|  | CHESS Score 3 | 7.2 | (2.5, 11.9) | 12.4 | (6.6, 18.3) |
|  | CHESS Score 4 | 11.7 | (3.2, 20.1) | 14.5 | (2.6, 26.5) |
|  | ADLs 1st Quartile (≤ 17) | Reference |  |  |  |
|  | ADLs 2nd Quartile (18-19) | 0.7 | (-2.5, 3.8) | 5.9 | (3.5, 8.4) |
|  | ADLs 3rd Quartile (20-21) | 1.0 | (-2.3, 4.8) | 3.4 | (0.7, 6.1) |
|  | ADLs 4th Quartile (22-28) | 0.7 | (-3.5, 5.0) | 6.2 | (2.0, 10.3) |
|  | Acute Myocardial Infarction | 3.2 | (-0.4, 6.8) | 3.8 | (0.2, 7.4) |
|  | Alzheimer’s Disease / Dementia | -5.7 | (-9.2, -2.3) | -0.6 | (-3.6, 2.4) |
|  | Chronic Kidney Disease | 3.0 | (0.2, 5.8) | 8.5 | (6.0, 11.0) |
|  | Chronic Obstructive Pulmonary Disease | 4.9 | (2.3, 7.4) | 4.2 | (1.9, 6.5) |
|  | Ischemic Heart Disease | 2.8 | (-0.2, 5.7) | 1.4 | (-1.3, 4.0) |
|  | Hypertension | -8.5 | (-13.3, -3.7) | -5.9 | (-10.1, -1.7) |
| Facility-level Characteristics | Beds 1st Quartile (≤ 115 beds) | Reference |  |  |  |
|  | Beds 2nd Quartile (116 – 147 beds) | -3.8 | (-8.5, 0.9) | -1.4 | (-4.3, 1.5) |
|  | Beds 3rd Quartile (148 – 169 beds) | 0.1 | (-4.2, 4.4) | -2.1 | (-6.5, 2.3) |
|  | Beds 4th Quartile (≥ 170 beds) | -3.2 | (-7.5, 1.0) | 2.9 | (-6.6, 0.8) |
|  | Overall Star Rating 1 | 14.4 | (4.8, 24.0) | 11.8 | (6.3, 17.3) |
|  | Overall Star Rating 2 | 4.2 | (-0.8, 9.1) | 7.9 | (2.3, 13.6) |
|  | Overall Star Rating 3 | 1.8 | (-1.8, 5.5) | 2.0 | (-1.5, 5.5) |
|  | Overall Star Rating 4 | 2.8 | (-1.1, 6.6) | -0.4 | (-3.1, 2.2) |
|  | Overall Star Rating 5 | Reference |  |  |  |
|  | 2017 Quarter 2 | Reference |  |  |  |
|  | 2017 Quarter 3 | 0.2 | (-2.8, 3.2) | 1.7 | (-1.0, 4.4) |
|  | 2017 Quarter 4 | 2.0 | (-1.1, 5.1) | 2.6 | (-0.1, 5.3) |
|  | 2018 Quarter 1 | 2.0 | (-1.4, 5.2) | 3.3 | (0.5, 6.1) |
|  | 2018 Quarter 2 | 1.6 | (-5.7, 2.5) | 3.7 | (0.7, 6.6) |
| Abbreviations: ADLs = Activities of Daily Living, CHESS = Changes in Health, End-stage disease and Symptoms and Signs; Model also controls for residents with congestive heart failure, diabetes, depression, osteoporosis, stroke / transient ischemic attack and marital status, as well as facility characteristics staffing level, rural status, for-profit status, and multi-facility ownership. | | | | | |

Appendix 3. Estimated risk difference for characteristics associated with potentially avoidable hospitalizations, stratified by clinical + payment and payment only facilities.

|  |  | **Potentially Avoidable Hospitalizations (n=487)** | | | |
| --- | --- | --- | --- | --- | --- |
|  |  | **Clinical + Payment (n=5,555 resident-quarters)** | | **Payment Only**  **(n=7,232 resident-quarters)** | |
| Individual-level Characteristics | Aged ≤ 64 years | 1.7 | (-0.1, 3.5) | 0.5 | (-1.7, 2.8) |
|  | Aged 65-74 years | Reference |  |  |  |
|  | Aged 75-84 years | -0.1 | (-1.6, 1.4) | 0.2 | (-1.4, 1.9) |
|  | Aged 85-94 years | -2.1 | (-3.8, -0.3) | -1.0 | (-2.6, 0.7) |
|  | Aged ≥ 95 years | -6.2 | (-10.4, -2.0) | -1.7 | (-3.7, 0.3) |
|  | Female | 0.3 | (-0.9, 1.5) | -0.1 | (-1.3, 1.0) |
|  | White | Reference |  |  |  |
|  | Black | 0.9 | (-0.5, 2.2) | 3.4 | (0.4, 6.5) |
|  | Other | 1.4 | (-2.1, 4.8) | -0.8 | (-4.5, 3.0) |
|  | Dual Eligible | -3.1 | (-4.3, -1.8) | -2.1 | (-3.1, -1.0) |
|  | Cognitive Functioning Scale 1 | Reference |  |  |  |
|  | Cognitive Functioning Scale 2 | -1.3 | (-2.9, 0.1) | 0.4 | (-1.1, 1.9) |
|  | Cognitive Functioning Scale 3 | -0.6 | (-2.0, 0.8) | -1.7 | (-3.1, -0.4) |
|  | Cognitive Functioning Scale 4 | -2.6 | (-5.2, -0.1) | -4.2 | (-5.5, -2.9) |
|  | CHESS Score 0 | Reference |  |  |  |
|  | CHESS Score 1 | 1.4 | (-0.1, 3.0) | 0.9 | (-0.2, 1.9) |
|  | CHESS Score 2 | 2.0 | (0.3, 3.8) | 2.9 | (1.3, 4.6) |
|  | CHESS Score 3 | 3.9 | (1.6, 6.2) | 4.3 | (0.9, 7.7) |
|  | CHESS Score 4 | 6.1 | (2.3, 9.9) | 7.2 | (-1.4, 15.8) |
|  | ADLs 1st Quartile (≤ 17) | Reference |  |  |  |
|  | ADLs 2nd Quartile (18-19) | 0.5 | (-1.0, 2.0) | 1.0 | (-0.2, 2.2) |
|  | ADLs 3rd Quartile (20-21) | -1.1 | (-2.7, 0.5) | 0.4 | (-0.9, 1.7) |
|  | ADLs 4th Quartile (22-28) | -1.4 | (-3.5, 0.6) | 1.1 | (-0.9, 3.1) |
|  | Acute Myocardial Infarction | -0.1 | (-1.7, 1.6) | 2.5 | (1.0, 3.9) |
|  | Alzheimer’s Disease / Dementia | 0.1 | (-1.5, 1.8) | 0.6 | (-0.7, 2.0) |
|  | Chronic Kidney Disease | 0.3 | (-1.1, 1.7) | 2.5 | (1.2, 3.8) |
|  | Chronic Obstructive Pulmonary Disease | 1.2 | (-0.1, 2.4) | 0.9 | (-0.2, 2.0) |
|  | Ischemic Heart Disease | 1.2 | (-0.3, 2.6) | 1.3 | (-0.1, 2.7) |
|  | Hypertension | -4.0 | (-6.3, -1.6) | -3.7 | (-5.8, -1.6) |
| Facility-level Characteristics | Beds 1st Quartile (≤ 115 beds) | Reference |  |  |  |
|  | Beds 2nd Quartile (116 – 147 beds) | -2.7 | (-5.0, -0.5) | 0.4 | (-1.0, 1.7) |
|  | Beds 3rd Quartile (148 – 169 beds) | -0.5 | (-2.5, 1.5) | 1.4 | (-1.0, 3.9) |
|  | Beds 4th Quartile (≥ 170 beds) | -1.9 | (-3.9, 0.2) | -0.6 | (-2.3, 1.2) |
|  | Overall Star Rating 1 | 2.3 | (-1.9, 6.6) | 0.6 | (-2.0, 3.2) |
|  | Overall Star Rating 2 | 0.4 | (-2.0, 2.9) | -0.6 | (-3.1, 1.8) |
|  | Overall Star Rating 3 | -0.5 | (-2.3, 1.4) | -1.6 | (-3.2, 0.1) |
|  | Overall Star Rating 4 | 0.3 | (-1.6, 2.3) | -0.9 | (-2.3, 0.5) |
|  | Overall Star Rating 5 | Reference |  |  |  |
|  | 2017 Quarter 2 | Reference |  |  |  |
|  | 2017 Quarter 3 | 1.1 | (-0.4, 2.7) | 1.1 | (-0.2, 2.4) |
|  | 2017 Quarter 4 | 0.4 | (-1.3, 2.1) | 1.4 | (0.1, 2.8) |
|  | 2018 Quarter 1 | 0.6 | (-1.2, 2.4) | 1.5 | (0.1, 2.9) |
|  | 2018 Quarter 2 | 1.0 | (-1.1, 3.1) | 1.5 | (0.1, 3.0) |
| Abbreviations: ADLs = Activities of Daily Living; CHESS = Changes in Health, End-stage disease and Symptoms and Signs; Model also controls for residents with congestive heart failure, diabetes, depression, osteoporosis, stroke / transient ischemic attack and marital status, as well as facility characteristics staffing level, rural status, for-profit status, and multi-facility ownership. | | | | | |
